# Retropharyngeal course of the superior thyroid artery – a novel finding

**DOI:** 10.1007/s00276-025-03627-7

**Published:** 2025-04-03

**Authors:** Rodica Narcisa Calotă, Mugurel Constantin Rusu, Cătălin Constantin Dumitru, Liliana Moraru, Răzvan Costin Tudose

**Affiliations:** 1 Division of Anatomy, Faculty of Dentistry, “Carol Davila” University of Medicine and Pharmacy, Bucharest, RO-020021 Romania; 2 Department of Oral and Maxillofacial Surgery, “Carol Davila” Central Military Emergency Hospital, Bucharest, Romania; 3 Faculty of Dentistry, “Titu Maiorescu” University, Bucharest, Romania; 4 Division of Anatomy, Faculty of Dentistry, Carol Davila University of Medicine and Pharmacy, Bucharest, 050474 Romania

**Keywords:** Carotid artery, Computed tomography, Larynx, Pharynx, Thyroid gland

## Abstract

**Purpose:**

The anatomical variables of the superior thyroid artery (STA) are well-studied. It typically leaves the external carotid artery (ECA) and descends on the inferior pharyngeal constrictor muscle to reach the thyroid lobe. We serendipitously found a novel possibility: the bilateral retropharyngeal course of the STA, which we report here.

**Method:**

The case was found while studying the archived angioCT file of a 56-year-old male.

**Results:**

The right carotid bifurcation (CB) was in the coronal plane at 3.3 mm inferior to the greater horn of the hyoid bone (GHHB). The initial segment of the ECA was medial to the GHHB. The origin of the right STA was at 2.7 cm medial to the GHHB greater hyoid horn from the anterior side of the ECA. The left CB was at 2.5 mm posterior to the left hyoid tubercle. It was oriented sagittally oblique, with the left ECA antero-medially to the left ICA. The left STA arose from the medial side of the ECA at 5.6 mm postero-superior to the hyoid tubercle. Each STA descended medially to the GHHB and, further, the superior horn of the thyroid cartilage on that side. At the root of the superior horn of the thyroid cartilage, each STA turned laterally between the common carotid artery and the posterior margin of the lamina of the thyroid cartilage and continued to the thyroid lobe on that side. Thus, both STAs coursed posteriorly to the pyriform recess of the hypopharynx on that side.

**Conclusion:**

Finding bilateral STAs is extraordinary but possible. Such extremely rare variants can be accurately identified during preoperative angioCT scans.

## Introduction

The thyroid arteries supply the thyroid gland. The superior thyroid artery (STA) is typically a branch of the external carotid artery (ECA) [7]. It may also arise from the common carotid artery (CCA) or the carotid bifurcation (CB). It usually originates inferiorly to the greater horn of the hyoid bone (GHHB) and descends on the lateral pharyngeal wall, in the carotid triangle, then beneath the superior belly of the omohyoid muscle and reaches the thyroid lobe. Previous studies evaluated the STA’s origin from the carotid axis, the common trunks it may form, or different branching patterns [2, 11]. To our knowledge, a retropharyngeal course of the STA has not been reported previously. Therefore, we aimed to report such a novel variant found bilaterally during an angioCT anatomical study.

## Materials and methods

Determinations were performed using archived angioCT imaging data from a 56-year-old adult male. The scan was adequate, and there were no pathological processes distorting the vascular anatomy in the neck. The research followed the principles of the World Medical Association Code of Ethics (Declaration of Helsinki). The responsible authorities (affiliation 2) approved the study (approval no. 437/2021).

The CT scans were performed as previously with a 32-slice scanner (Siemens Multislice Perspective Scanner, Forcheim, Germany), with a 0.6 mm collimation and a reconstruction of 0.75 mm thickness, with 50% overlap for a multiplanar maximum intensity projection [3]. We used the Horos for macOS (Horos Project) program, as in previous studies [5]. Findings were documented on two-dimensional slices and three-dimensional volume renderings.

## Results

**Fig. 1:**
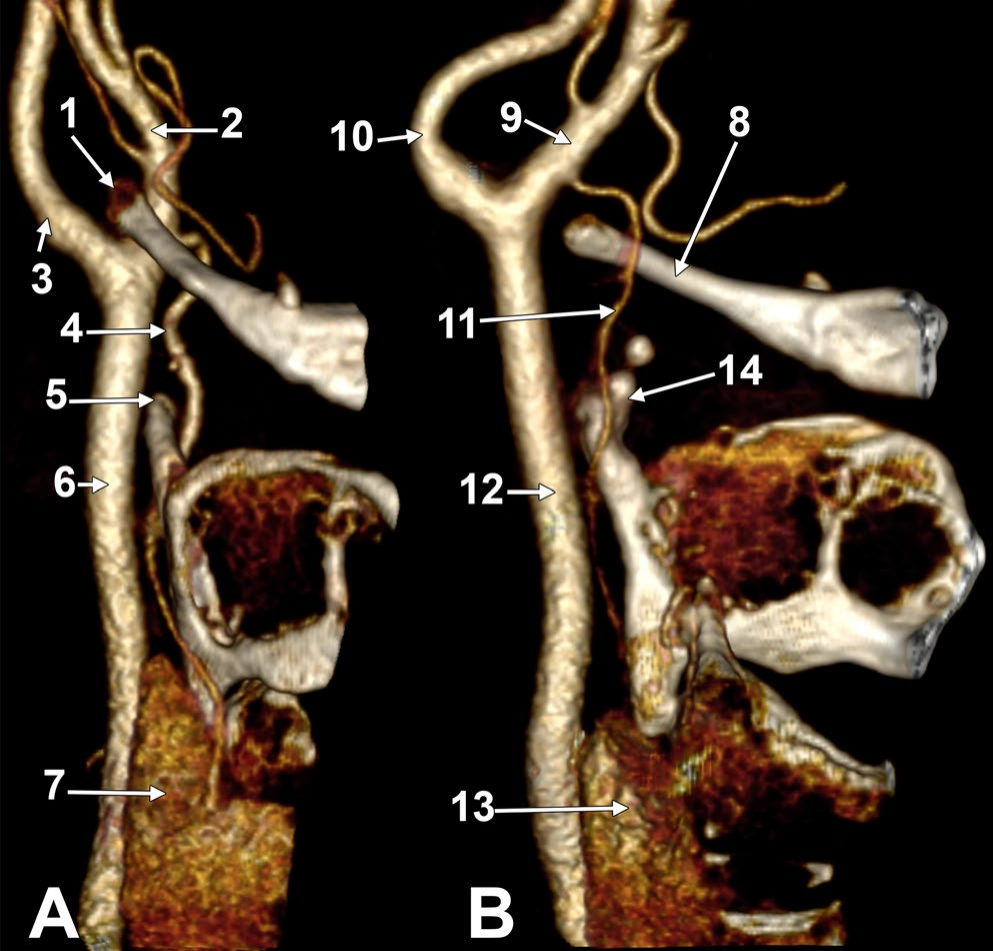
Three-dimensional renderings: **A**. Right side, anterollateral view. **B**. Left side, medial view. (1) right hyoid tubercle; (2) right external carotid artery; (3) right internal carotid artery; (4) right superior thyroid artery; (5) right superior horn of the thyroid cartilage; (6) right common carotid artery; (7) right thyroid lobe; (8) left greater hyoid horn; (9) left external carotid artery; (10) left internal carotid artery; 11. left superior thyroid artery; 12. left common carotid artery; 13. left thyroid lobe; 14. left superior horn of the thyroid cartilage

The CB on the right side was in the coronal plane at 3.3 mm inferior to the GHHB. The right hyoid tubercle was penetrating between the ICA and the ECA. The ICA was postero-lateral to the GHHB and the ECA. So, the initial segment of the ECA was medial to the GHHB. The origin of the right STA was at 2.7 cm medial to the GHHB, from the anterior side of the ECA. It continued inferiorly on the medial side of the superior horn of the thyroid cartilage. At the root of the superior horn of the thyroid cartilage, it turned infero-laterally. It crossed between the posterior margin of the lamina of the thyroid cartilage and the CCA and continued to the right thyroid lobe (Fig. 1A).

The left CB was at 2.5 mm posterior to the left hyoid tubercle. It was oriented sagittally oblique, with the left ECA antero-medially to the left ICA. The left STA arose from the medial side of the ECA at 5.6 mm postero-superior to the hyoid tubercle. Then, it continued inferiorly on the medial side of the GHHB and superior horn of the thyroid cartilage. At the root of the superior horn of the thyroid cartilage, it turned laterally between the CCA and the posterior margin of the lamina of the thyroid cartilage and continued to the left thyroid lobe. (Fig. 1B).

**Fig. 2:**
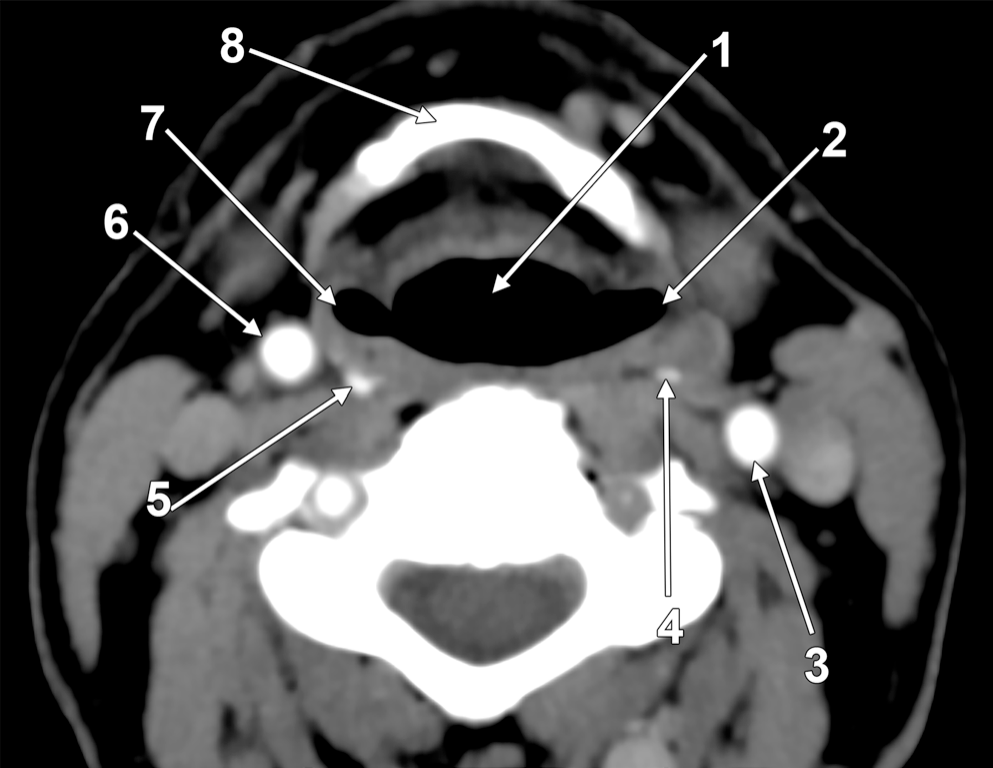
Inferiorly viewed axial slice through the retropharyngeal superior thyroid arteries. (1) hypopharynx; (2) left pyriform recess; (3) left common carotid artery; (4) left superior thyroid artery; (5) right superior thyroid artery; (6) right common carotid artery; (7) right pyriform recess; (8) hyoid bone

Therefore, both STAs initially coursed on the posterior walls of the pyriform recesses of the pharynx at the level of the C5-C6 cervical vertebrae (Fig. 2).

## Discussion

According to different publications, the origin of the STA is infrahyoid [4]. We found here the right STA originating at the level of the GHHB and the left one with suprahyoid origin. No similar findings were reported to our knowledge. A hyoid or suprahyoid level of the origin of the STA is an unexpected finding that alters the typical value as a landmark of the GHHB.

A case was described in which the STA originated from the right ascending pharyngeal artery (APA) [10]. The APA arose from the medial aspect of the ECA. The STA had two segments: a horizontal segment traveling postero-medially to the linguofacial trunk, directed anteriorly, medially, and slightly inferiorly; and a descending segment moving inferiorly, anteriorly, and medially towards the superior pole of the thyroid gland [10]. This descending segment passed posterior to the superior laryngeal artery and a muscular branch to the infrahyoid muscles originating directly from the ECA [10]. Although not explicitly described as retropharyngeal, the STA’s origin and course may suggest proximity to the retropharyngeal space.

Another case report described the STA originating from the internal carotid artery (ICA) via a common trunk shared with the APA and occipital arteries [1]. Although this anatomical variation might suggest a more profound trajectory of the STA, the exact courses of the ICA and STA were not detailed, leaving any relationship to the retropharyngeal space speculative.

The retropharyngeal space is a critical anatomical space which does not contain major arteries. However, it is well known that the ICA may have a retropharyngeal deviated course [6, 8, 9]. Also, the common carotid artery, the CB and the ECA may reach within the retropharyngeal space [8]. High retropharyngeal arteries may be injured during pharynx surgery, tonsillectomy, peritonsillar abscess drainage, or transoral tumor resection [8].

Here, we report STAs coursing posteriorly to the pyriform recesses of the hypopharynx and further crossing the posterior margin of the thyroid cartilage lamina. These aberrant courses make the STAs prone to injury during surgical approaches to the pyriform recess, hypopharynx or larynx.

## Conclusions

Finding bilateral retropharyngeal STAs is extraordinary but possible. Such extremely rare variants can be accurately identified during preoperative angioCT scans.

## Data Availability

No datasets were generated or analysed during the current study.
